# A comparative study of Kirschner wire intramedullary fixation versus bicortical fixation for metaphyseal-diaphyseal junction fractures of the pediatric distal radius

**DOI:** 10.1186/s12891-026-10316-y

**Published:** 2026-08-04

**Authors:** Zhenhua Xu, Yancai Yang, Qi Chen

**Affiliations:** Department of Pediatric Surgery, Affiliated Women and Children’s Hospital of Ningbo University, No.339 Liuting Street, Ningbo, Zhejiang 315012 China

**Keywords:** Pediatric radius fractures, Kirschner wire, Intramedullary fixation, Bicortical fixation

## Abstract

**Background:**

This study compared the clinical efficacy of closed reduction with Kirschner wire (K-wire) intramedullary fixation versus bicortical fixation for pediatric distal radius metaphyseal-diaphyseal junction (MDJ) fractures.

**Methods:**

We retrospectively analyzed 61 children with closed distal radius MDJ fractures admitted to the Affiliated Women and Children’s Hospital of Ningbo University between May 2021 and November 2024. Patients were divided into two groups according to the K-wire fixation method: intramedullary fixation group (IFG, 32 cases; K-wire without contralateral cortical penetration) and bicortical fixation group (BFG, 29 cases; K-wire with contralateral cortical penetration). We compared surgical time, fracture healing time, 6-month postoperative wrist function, and complication rates between groups.

**Results:**

All 61 procedures achieved successful closed reduction; mean follow-up was 15.3 months (range: 6–24 months). Baseline data were comparable between groups. Surgical time did not differ significantly. Fracture healing time was shorter in the IFG. There were no significant differences in 6-month postoperative wrist function or overall complication rate between groups, although the complication rate was markedly lower in the IFG. No vascular, nerve, or tendon injuries, nonunions, or premature physeal closures occurred in either group during the follow-up period.

**Conclusions:**

Closed reduction with K-wire fixation is an effective treatment for pediatric distal radius MDJ fractures. Compared with bicortical K-wire fixation, intramedullary K-wire fixation offers advantages including shorter fracture healing time and a trend toward a lower complication rate.

## Introduction

Distal radius fractures are common in children, second only to supracondylar humeral fractures [1–4]. Among these, metaphyseal fractures of the distal radius are the most common. The primary treatment involves closed reduction followed by plaster or splint fixation. However, surgical intervention becomes necessary for fractures that are difficult to reduce or if redisplacement occurs after conservative treatment [5, 6]. In such cases, percutaneous Kirschner wire (K-wire) fixation is the gold standard for the surgical management of pediatric distal radius metaphyseal fractures [7].

Distal radius metaphyseal-diaphyseal junction (MDJ) fractures present a unique challenge compared to distal radius metaphyseal fractures due to their specific anatomical characteristics. Improper treatment can lead to complications such as fracture redisplacement and nonunion [8, 9]. Currently, there is no standardized surgical fixation method for these fractures. Various techniques have been reported, including the use of elastic stable intramedullary nails (ESIN) [10–13], K-wires [14], plates [15], and external fixators [16].

This study retrospectively analyzed clinical data from 61 pediatric patients with distal radial MDJ fractures who met the inclusion criteria and were admitted to the Affiliated Women and Children’s Hospital of Ningbo University between May 2021 and November 2024. The patients were divided into two groups: one underwent K-wire intramedullary fixation, while the other received K-wire bicortical fixation. The study aimed to compare and analyze the efficacy of these two treatment methods.

## Methods

### General information

**Fig. 1:**
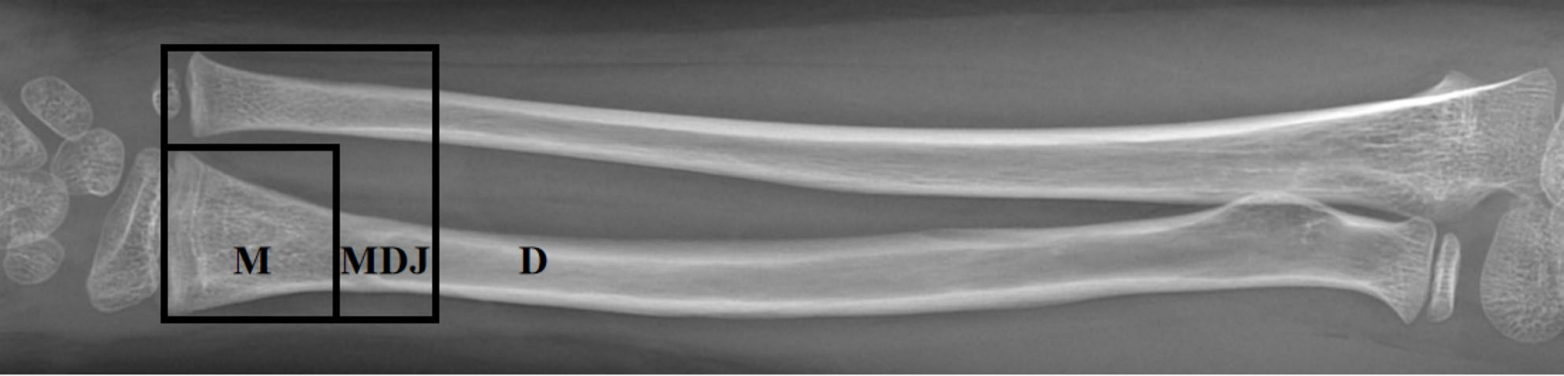
Definition of the distal radial metaphyseal-diaphyseal junction (MDJ). M = metaphysis of the distal radius; MDJ = metaphyseal-diaphyseal junction; D = diaphysis of the radius

A retrospective analysis examined the clinical data of 61 pediatric patients with distal radial MDJ fractures treated surgically at the Affiliated Women and Children’s Hospital of Ningbo University between May 2021 and November 2024. In this study, the distal radial MDJ was defined according to the Lieber method [17]: On standard anterior–posterior radiographic view of the forearm, two squares were constructed. The smaller square had a side length equal to the width of the distal radial physis, while the larger square had a side length equal to the combined width of the distal radial and ulnar physes. The non-overlapping area between the two squares was defined as the distal radial MDJ (Fig. 1). All fractures were closed. The cohort included 51 males and 10 females, with a mean age of 8.7 years (range 2.3–13.7 years). The injuries affected the left side in 32 cases and the right side in 29 cases. Based on the injury mechanism and displacement direction of the distal fracture fragment, fractures were categorized as either extension type (fall on an outstretched palm with dorsal displacement of the distal fragment, 40 cases) or flexion type (fall on the dorsum of the hand with volar displacement of the distal fragment, 21 cases), determined by specific imaging features. 5 patients had isolated radial fractures, and 56 had concomitant distal ulnar fractures. The median time from injury to surgery was 3.3 days (range 1–22 days). Ulnar fixation was performed in 27 cases.

Inclusion criteria: (1) Patients younger than 14 years; (2) Distal radial MDJ fracture confirmed by radiography, with unsuccessful closed reduction or redisplacement after conservative treatment; (3) Written informed consent from the patient’s guardians.

Exclusion criteria: (1) Galeazzi fracture-dislocations; (2) Open fractures; (3) Fractures resulting from high-energy trauma, such as traffic accidents or falls from heights; (4) Follow-up periods shorter than 6 months.

All fractures underwent successful treatment through closed reduction. Transphyseal K-wires were used for internal fixation of the radius. Based on the fixation method, patients were divided into two groups: the intramedullary fixation group (IFG, 32 cases, K-wire without contralateral cortical penetration) and the bicortical fixation group (BFG, 29 cases, K-wire with contralateral cortical penetration). Baseline data showed no statistically significant differences between the groups (*P* > 0.05, Table 1), ensuring good comparability.

The Medical Ethics Committee of the Affiliated Women and Children’s Hospital of Ningbo University approved this study (Approval No.: NBFE-2025-KY-153).

### Surgical methods

#### IFG

Under intravenous general anesthesia or brachial plexus block, routine disinfection and draping are performed. The fracture is reduced under C-arm fluoroscopic guidance, utilizing K-wire joysticking if necessary. Once reduction is satisfactory, a 1.6-mm or 2.0-mm K-wire is percutaneously inserted from both the radial styloid process and the dorsal aspect of the lunate fossa, crossing the fracture line into the proximal medullary canal without penetrating the contralateral cortex. C-arm fluoroscopy confirms satisfactory fracture reduction, proper implant positioning, and unimpeded passive wrist motion. The K-wires are then bent, cut, and left protruding percutaneously. Concomitant distal ulnar fractures with significant displacement and instability after reduction are fixed using ESIN or K-wires. The wire ends are covered with iodophor gauze, and the forearm is immobilized in a plaster cast postoperatively.

#### BFG

Under the same anesthesia, the affected limb was disinfected and draped. A closed reduction of the fracture was performed using C-arm fluoroscopic guidance, with K-wire joysticking applied if needed. A 1.6-mm or 2.0-mm K-wire was then percutaneously inserted from both the radial styloid process and the dorsal aspect of the lunate fossa. These wires crossed the fracture line and penetrated the contralateral cortex. Fluoroscopy confirmed satisfactory fracture reduction, proper implant positioning, and unrestricted passive wrist motion. The K-wires were bent, cut, and left protruding percutaneously. For distal ulnar fractures with significant displacement and instability, ESIN or K-wires were used for fixation. The exposed wire ends were covered with iodophor gauze, and the forearm was immobilized in a plaster cast postoperatively.

**Table 1:** Baseline data of the two groups of patients

Item	IFG (*n* = 32)	BFG (*n* = 29)	t/Z/χ² Value	*P* Value
Age (y, mean ± SD)	8.3 ± 2.7	9.3 ± 2.9	-1.477	0.145
Gender [*n* (% )]			0.031	0.860
Male	26(81.2)	25(86.2)		
Female	6(18.8)	4(13.8)		
Side [*n* (% )]			0.012	0.913
Left	17(53.1)	15(51.7)		
Right	15(46.9)	14(48.3)		
Fracture type [*n* (% )]			0.301	0.583
Extension	22(68.8)	18(62.1)		
flexion	10(31.2)	11(37.9)		
Concomitant Ulnar Fracture [*n* (% )]			0.013	0.909
Yes	30(93.8)	26(89.7)		
No	2(6.2)	3(10.3)		
Time from Injury to Surgery (d, median [Q1, Q3])	2.5[1,4]	2[1,3.5]	-0.06	0.952
Ulnar fixation [*n* (% )]			1.248	0.264
Yes	12(37.5)	15(51.7)		
No	20(62.5)	14(28.3)		

Independent samples t-test was used for age; the Mann–Whitney U test for time from injury to surgery; Pearson chi-square test for side, fracture type, and ulnar fixation; and continuity-corrected chi-square test for gender and concomitant ulnar fracture

### Postoperative management and follow-up

After surgery, patients received prophylactic antibiotics and anti-swelling treatment. Finger flexion-extension exercises began on the first postoperative day. Patients were discharged 3 to 5 days post-surgery after a dressing change.

Outpatient follow-up involved weekly dressing changes to monitor for pin-tract infections, pin-end irritation and loosening or failure of internal fixation. Biweekly X-rays were taken to assess fracture healing by observing callus formation. Clinical union was confirmed when 3-directional bridging callus appeared at the fracture ends. The plaster cast and internal fixation were removed and wrist function exercises were started.

Six months after surgery, wrist function was evaluated using the Gartland-Werley score [18], which assesses subjective symptoms, global wrist range of motion, residual deformity, and postoperative complications. Clinical outcomes were graded as excellent (0–2 points), good (3–8 points), fair (9–20 points), and poor (≥ 21 points). All assessments were performed by a single attending orthopedic surgeon who was not involved in patient treatment.

### Statistical Methods

Statistical analysis was performed with IBM SPSS 27.0. Categorical variables were summarized as numbers and percentages, and group comparisons were made using the Pearson chi-square test, continuity-corrected chi-square test, or Fisher’s exact test where appropriate. For ordinal data such as Gartland-Werley wrist function scores, group differences were analyzed with the Mann-Whitney U test. The Shapiro-Wilk test was used to check whether measurement data were normally distributed, and the Levene test was applied to test homogeneity of variance for normally distributed variables. When both conditions were satisfied, data were expressed as mean ± SD, and inter-group comparisons were conducted using the independent samples t-test. For non-normally distributed data, results were expressed as median [Q1, Q3], with comparisons made using the Mann-Whitney U test. To control type I error inflation, a Bonferroni correction was applied for multiple comparisons of four outcome measures, setting the adjusted significance level at α = 0.0125. A P value of less than 0.0125 was deemed statistically significant. Baseline characteristics were compared at an unadjusted α = 0.05 level. Post-hoc power analysis was performed using G*Power 3.1 software at a conventional significance level of α = 0.05, with exact power calculated via Fisher’s exact test for complication rate comparisons. In addition to P-value reporting, we provided the effect size (Phi coefficient), risk difference (RD), and corresponding 95% confidence intervals (CI). Fracture healing time, considered as time-to-event data, was expressed as median (95% confidence interval [95% CI]) and analyzed using the Kaplan-Meier method. Differences in healing time between the two groups were compared using the log-rank test.

## Results

All 61 patients successfully underwent closed reduction. The follow-up period exceeded 6 months, averaging 15.3 months, with a range of 6 to 24 months.

Surgical time for the Intramedullary Fixation Group (IFG) was 41 [35, 63] minutes, while for the Bicortical Fixation Group (BFG), it was 46 [30, 68] minutes. There was no significant difference between the two groups (*P* > 0.0125, Table 2).

**Table 2:** Comparison of surgical time and fracture healing time between groups

Item	IFG (*n* = 32)	BFG (*n* = 29)	Z / χ² Value	*P* Value
Surgical Time (min, median [Q1, Q3])	41 [35,63]	46 [30,68]	-0.347	0.729
Fracture Healing Time [w, median (95% CI)]	5.9 (5.7–6.1)	6.9 (6.4–7.4)	9.107	0.003

Mann-Whitney U test was used for surgical time, and log-rank test (Kaplan-Meier survival analysis) for fracture healing time

The fracture healing time for IFG was 5.9 (5.7–6.1) weeks, significantly shorter than the 6.9 (6.4–7.4) weeks observed for BFG (*P* < 0.0125, Table 2).

Six months after surgery, wrist function outcomes were as follows: in the IFG group, 29 were rated excellent, 3 good, and none fair. In the BFG group, 24 were excellent, 4 good, and 1 fair. There was no significant difference between the groups (*P* > 0.0125, Table 3).

**Table 3:** Comparison of wrist function at 6 months postoperatively between groups

Item	IFG (*n* = 32)	BFG (*n* = 29)	Z Value	*P* Value
Wrist Function (Gartland-Werley Score, *n*)			0.937	0.349
Excellent	29	24		
Good	3	4		
Fair	0	1		
Poor	0	0		

Wrist function was graded by the Gartland-Werley score and compared with the Mann-Whitney U test

The complication rate was 6.3% (2 out of 32) for IFG, which was lower than 13.8% (4 out of 29) for BFG. However, this difference was not statistically significant (continuity-corrected chi-square test, *P* > 0.0125, Table 4). The RD was − 7.5% with a 95% CI ranging from − 22.6% to 7.6%, and the Phi coefficient was 0.13. The post-hoc statistical power was 0.14. During follow-up, there were no occurrences of vascular, nerve, or tendon injuries, nonunion, or premature physeal closure. Pin tract infections were reported in 2 cases each for IFG and BFG, all of which were successfully treated with intensified dressing changes and oral antibiotics. In the BFG group, 1 case of fracture displacement and 1 case of refracture were noted; both were resolved with closed reduction and plaster casting.

**Table 4:** Comparison of postoperative complication rate between groups

Item	IFG (*n* = 32)	BFG (*n* = 29)	χ² Value	*P* Value	RD (95% CI)	Effect size (Phi)
Complication Rate [*n* (%)]	2(6.3%)	4(13.8%)	0.311	0.577	−7.5% (− 22.6% to 7.6%)	0.13

Complication rate was compared using the continuity-corrected chi-square test

*RD* Risk difference, *CI* confidence interval

**Fig. 2:**
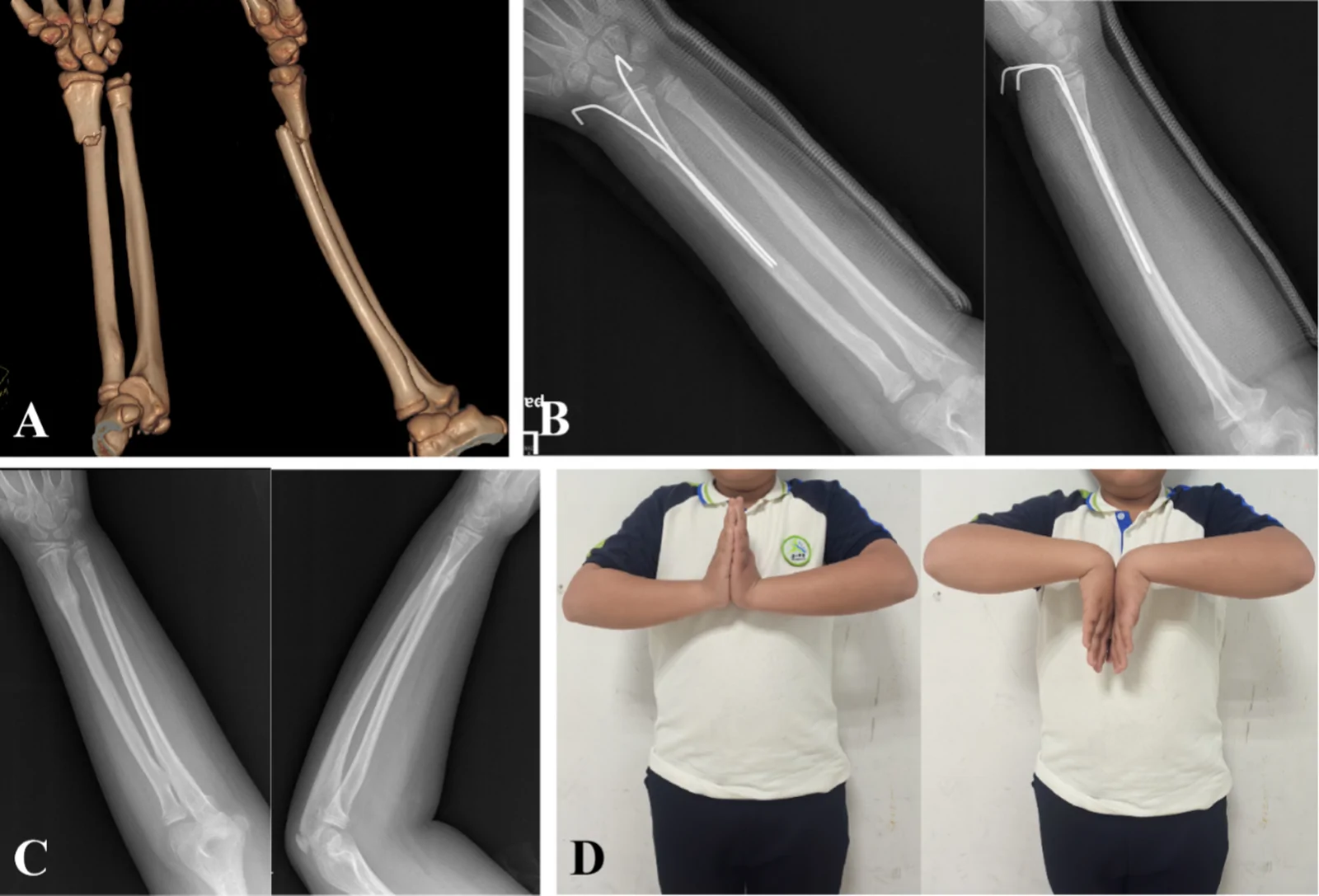
**A** Preoperative CT scan; **B** Postoperative day 1 radiograph; **C** Radiograph after Kirschner wire removal; **D** Clinical photograph of wrist function at 6 months postoperatively

**Fig. 3:**
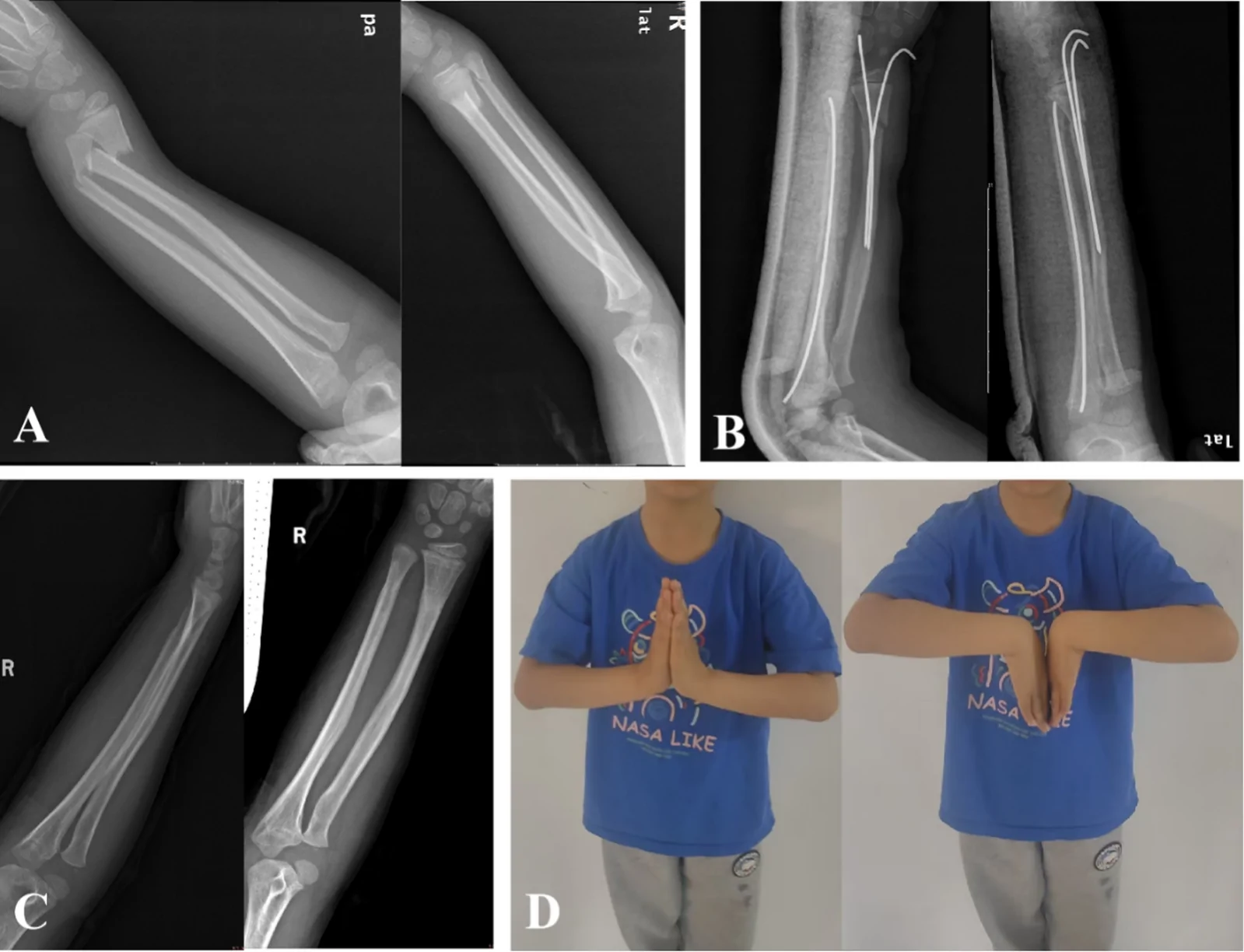
**A** Preoperative radiograph; **B** Postoperative day 1 radiograph; **C** Radiograph after removal of all internal fixations; **D** Clinical photograph of wrist function at 6 months postoperatively

**Fig. 4:**
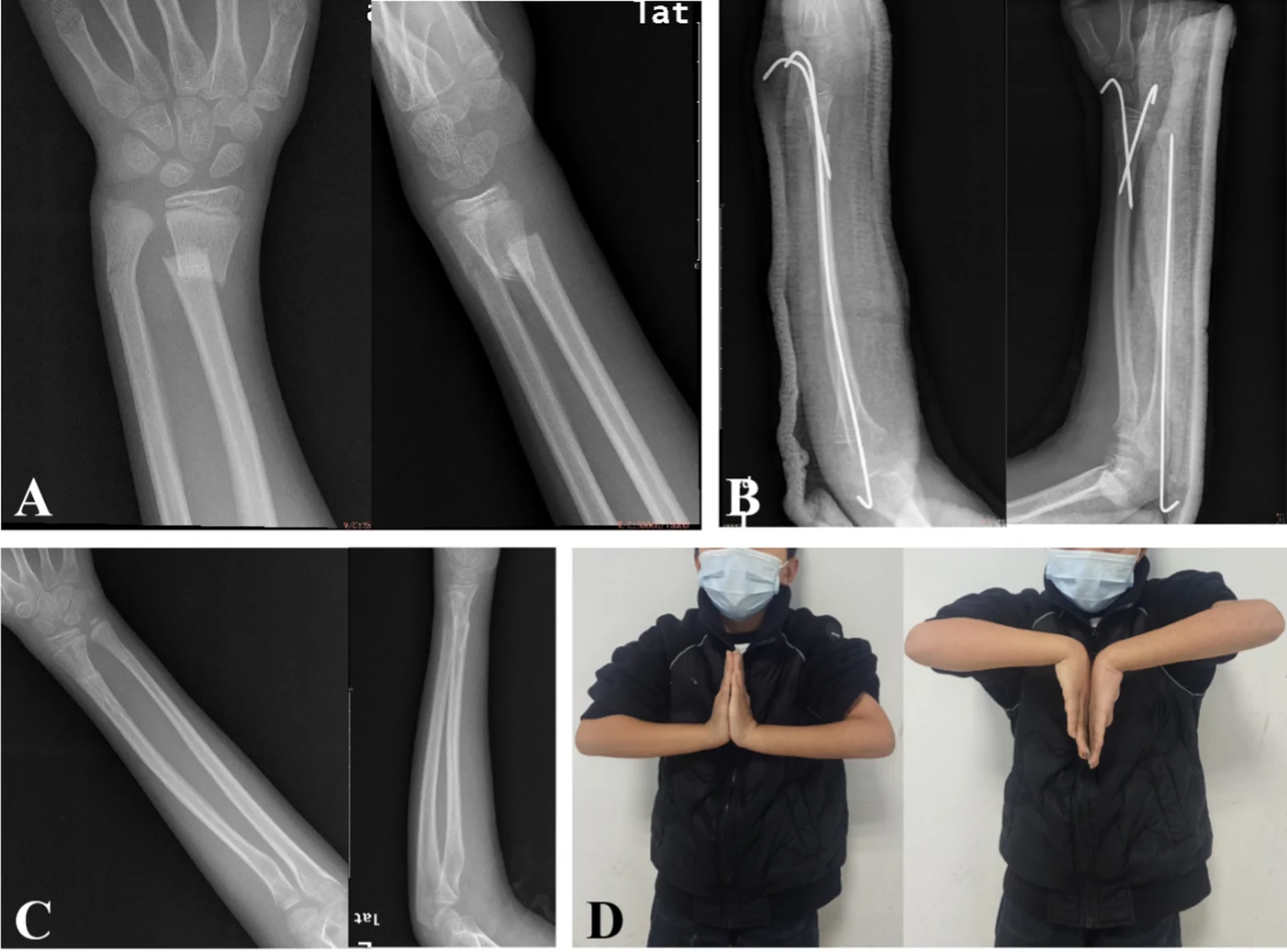
**A** Preoperative radiograph; **B** Postoperative day 1 radiograph; **C** Radiograph after removal of all internal fixations; **D** Clinical photograph of wrist function at 6 months postoperatively

Typical cases are shown in Figs. 2, 3 and 4.

## Discussion

Distal metaphyseal-diaphyseal junction (MDJ) fractures of the radius in children represent a distinct type of distal radial fracture. In 2010, Lieber et al. noted that fractures at the transition zone between the metaphysis and diaphysis tend to be unstable. They identified this region as the MDJ [17]. This area has a less robust blood supply compared to the metaphysis. Consequently, hematoma formation is slower or more difficult following MDJ fractures, which can delay the healing process. Anatomically, the distal radial metaphysis is flat and wide, whereas the diaphysis is tubular. The medullary cavity at the MDJ widens distally toward the metaphysis and narrows proximally toward the diaphysis. This anatomical variation complicates fracture fixation. Poor reduction or inadequate fixation strength increases the likelihood of displacement due to muscle and ligament traction. Consequently, MDJ fractures are more prone to delayed healing, nonunion, or redisplacement than typical metaphyseal fractures. Literature indicates that most cases of nonunion in children’s distal radial fractures occur at the MDJ [19]. Therefore, achieving good reduction and stable fixation is crucial to minimizing complications.

For stable MDJ fractures of the distal radius in children, conservative treatment is recommended as the primary approach [20]. In our clinical practice, we found that manual reduction followed by a forearm U-shaped cast for 4-6 weeks generally yields satisfactory outcomes. However, the stability of MDJ fractures in children post-reduction is inferior to that of metaphyseal fractures [21]. Proctor et al. reported a 52% redisplacement rate after the reduction of initially completely displaced distal radial fractures in children, with a 23% redisplacement rate following re-reduction [22]. Additionally, repeated forceful manual reduction during the acute phase poses a risk of forearm compartment syndrome. Consequently, for MDJ fractures that are completely displaced and exhibit redisplacement after conservative treatment, we advocate for surgical intervention. This recommendation aligns with the guidelines for treating completely displaced MDJ fractures of the distal radius in children as outlined in the third edition of "AO Treatment of Fractures."

There remains no uniform surgical approach for pediatric distal radial MDJ fractures, as each fixation method carries distinct benefits and drawbacks.

Markus Dietzel and colleagues [23] treated 20 patients with unstable distal radial MDJ using antegrade ESIN. The procedure was simple, safe and reliable and allowed full recovery of forearm range of motion. The authors recommended immediate postoperative rehabilitation without external immobilization. However, fixation with antegrade ESIN can cause injury to the deep branch of the radial nerve. To minimize irritation from the nail tail, it is often trimmed too short, which increases the technical difficulty of secondary extraction.

Jianyi J et al. [9] treated 25 pediatric patients with distal radial MDJ fractures using retrograde precision-shaping elastic intramedullary nailing (ESIN-RPS). Satisfactory clinical outcomes were achieved, although secondary surgery was required for implant removal.

Van Egmond and colleagues [24] treated 26 children with unstable distal radius fractures using open reduction and volar plate fixation. They had good functional and radiologic results. Plate internal fixation offers reduction and stable stabilization advantages, but the visible surgical scar often displeases children and their families and requires a second surgery.

Li et al. [16] conducted a study on 146 adolescent patients with radial MDJ fractures, comparing the use of non-bridging external fixators (EF) to crossed Kirsch-wire (K-wire) fixation. They found that the external fixator was more effective. It offered benefits such as shorter operative time, reduced tendon irritation, and improved radiographic outcomes. Despite these advantages, the cost-effectiveness of external fixators needs further investigation due to their higher expense compared to K-wire. An external fixator avoids the need for postoperative plaster cast immobilization, allowing early joint mobilization. However, external fixation is relatively expensive, technically demanding postoperatively, and associated with a risk of pin-tract infection.

Lieber et al. [17] described excellent outcomes in 10 children with distal radius MDJ fractures treated with transepiphyseal intramedullary K-wire fixation. Their results suggested that this technique holds clear advantages over other approaches for this particular fracture pattern. Our study yielded similar findings.

Wu R et al. [13] showed that both K-wire fixation and ESIN stabilize MDJ fractures of the pediatric distal radius. They noted that MDJ fractures are relatively far from the metaphysis, making it technically difficult to achieve sufficient bicortical fixation with K-wires. The steep implant angle and repeated adjustments of K-wire position may prolong operation time and raise the risk of physeal growth disturbances. In our study, the median surgical time for the BFG was 5 min longer than that for the IFG. While this difference did not reach statistical significance, our findings imply that bicortical K-wire fixation for MDJ fractures is technically more demanding. Such difficulty may become increasingly evident when the fracture line lies further away from the metaphysis.

Compared with other techniques, K-wire fixation for pediatric distal radius MDJ fractures carries several clear benefits: it is less costly, leaves smaller scars, and avoids the need for secondary surgery. These advantages make the procedure more acceptable to both children and their families.

K-wire fixation for distal radius MDJ fractures involves transphyseal fixation. Although the safety of smooth K-wires with a diameter of 2.0 mm or less crossing the physis has been well established [25], Giordano M et al. [26] evaluated 26 patients with forearm shaft fractures who underwent percutaneous transphyseal intramedullary K-wire fixation of the distal radius. Twelve months after K-wire removal, contrast-enhanced magnetic resonance imaging (MRI) of bilateral wrists showed no significant cartilage abnormalities related to the procedure in any patient. These findings align with our follow-up results, as none of the 61 cases in our study exhibited premature physeal closure during the follow-up period. However, repeated insertion of K-wires still poses a risk of iatrogenic premature physeal closure of the distal radius.

Older children often have strong muscle strength, making surgical reduction challenging, particularly in cases of simple radial fractures or greenstick fractures of the ulna. Closed reduction can be difficult under these circumstances. When fracture reduction and K-wire levering do not yield satisfactory results, open reduction becomes necessary. Although elastic fixation devices allow micromotion that can promote external callus bridging [27], K-wire fixation may not be ideal after open reduction due to potential damage to the periosteal blood supply. In such cases, plate internal fixation is recommended for enhanced stability.

In our study, closed reduction succeeded in all patients, and postoperative fracture union occurred in both groups. Wrist function recovery was satisfactory, and no neurovascular injuries were reported.

Although the median healing time for both groups was in the normal range (5.9 vs. 6.9 weeks), the difference was statistically significant after Bonferroni correction (*P* = 0.003). For pediatric patients, even a small reduction in healing time allows earlier return to daily activities, earlier removal of external fixation, and earlier return to sports. This improves patient satisfaction and lessens the burden imposed by casting or activity restrictions. We regard this difference as clinically relevant.

The complication rate was numerically lower in the IFG, indicating a potentially more favorable clinical course relative to the BFG.

Among minor complications, the most frequent were pin-site infections and skin irritation around the pin tails [28]. In this study, all four minor complications were superficial infections. These conditions resolved effectively with active management and did not affect the final surgical results.

Because there are few severe complications such as fracture displacement or fracture refracture, we are limited to descriptive data. In this study, two cases of severe postoperative complications occurred in the BFG. In the first case, a patient experienced plaster loosening and K-wire slippage three weeks postoperatively, with radiography confirming fracture displacement. The K-wires were removed, followed by manual reduction and additional plaster immobilization for five weeks. In the second case, a patient achieved clinical union six weeks postoperatively. However, one week after the removal of internal fixation, the patient suffered a refracture due to trauma, which was managed with manual reduction and plaster immobilization for five weeks. Ultimately, both patients achieved satisfactory fracture union and good recovery of wrist function.

Battle and colleagues [29] identified obvious biomechanical disparities between intramedullary and bicortical K-wire fixation when managing unstable forearm fractures in pediatric patients. In distal radial MDJ fractures, intramedullary K-wire placement can serve as an internal splint. It stabilizes the fracture site and aligns naturally with the mechanical axis of the radius. This fixation pattern allows appropriate physiological micromovement at the fracture interface, which promotes fracture healing. By comparison, bicortical fixation relies on cortical purchase. Such excessive stiffness inhibits favourable physiological motion at the fracture ends, inevitably slowing down the fracture healing process. When bicortical K-wire fixation is performed for MDJ fractures of the distal radius, the inherent limitation of the insertion angle often places the wire exit point close to the fracture site. This leads to inferior fixation stability relative to intramedullary fixation. We believe the occurrence of severe complications in the BFG is associated with the above factors.

The technical operation of K-wire intramedullary fixation is straightforward, yet certain details can impact surgical outcomes. We have outlined the following intraoperative precautions: (1) The K-wire must be inserted as perpendicular to the physis as possible. To prevent thermal injury, avoid prolonged high-speed drilling and minimize repeated passes to protect the physis. (2) Before cutting the wire, ensure that joint movement is unrestricted in wrist dorsiflexion and palmar flexion, and confirm there is no extensor tendon injury. A small incision at the pin insertion point can safely avoid tendons and nerves. (3) Leaving the K-wire tail outside the skin facilitates removal and prevents repeated friction that could injure the extensor pollicis longus tendon. Studies indicate that complication rates and functional outcomes are comparable to those of buried internal fixation [30]. (4) Bending the K-wire twice before cutting helps prevent skin injury from the pin tail, and reduces both skin irritation and pin tract infection. (5) Postoperative external fixation is important. A long-arm above-elbow plaster cast for 4–6 weeks is optimal. For older children with good compliance, a forearm plaster cast may be sufficient.

This study has several limitations. First, we used a retrospective design in which patients were grouped based on individual clinical data and intraoperative findings, rather than by randomization or predefined protocols. This may have led to selection bias between the groups. Second, our sample size was relatively small, with few complications observed, and the post-hoc statistical power was 0.14, which raises the risk of a false-negative Type II error. The nonsignificant difference in complication rates between groups should be considered cautiously, and intramedullary fixation may be superior. Third, our follow-up time was short, and longer follow-up is required to assess risks such as premature physeal closure. Future large-scale, prospective, multicenter comparative studies using propensity score matching are warranted.

## Conclusions

In summary, closed reduction with K-wire fixation yields favorable outcomes for distal radial MDJ fractures in children. The procedure is straightforward, avoids reoperation, minimizes surgical trauma, and achieves high parental satisfaction. Compared with bicortical K-wire fixation, intramedullary fixation shortens fracture healing time and has a numerically lower complication rate, indicating a more favorable clinical trend. Clinically, we recommend intramedullary fixation when K-wire fixation is indicated.

## Data Availability

The datasets used and/or analyzed during the current study are available from the corresponding author upon reasonable request.

## Abbreviations

K-wire: Kirschner wire
MDJ: Metaphyseal-diaphyseal junction
ESIN: Elastic stable intramedullary nail
IFG: Intramedullary fixation group
BFG: Bicortical fixation group
